# Regulation of Lymphatic GM-CSF Expression by the E3 Ubiquitin Ligase Cbl-b

**DOI:** 10.3389/fimmu.2018.02311

**Published:** 2018-10-08

**Authors:** Sebastian Peer, Giuseppe Cappellano, Natascha Hermann-Kleiter, Karin Albrecht-Schgoer, Reinhard Hinterleitner, Gottfried Baier, Thomas Gruber

**Affiliations:** ^1^Division of Translational Cell Genetics, Department for Medical Genetics, Molecular and Clinical Pharmacology, Medical University of Innsbruck, Innsbruck, Austria; ^2^Department of Dermatology, Venereology and Allergology, Medical University of Innsbruck, Innsbruck, Austria; ^3^Department of Immunology, University of Pittsburgh School of Medicine, Pittsburgh, PA, United States

**Keywords:** adaptive immunity, multiple sclerosis, experimental autoimmune encephalomyelitis, Cbl-b, GM-CSF

## Abstract

Genome-wide association studies as well as lymphatic expression analyses have linked both Cbl-b and GM-CSF to human multiple sclerosis as well as other autoimmune diseases. Both Cbl-b and GM-CSF have been shown to play a prominent role in the development of murine encephalomyelitis; however, no functional connection between the two has yet been established. In this study, we show that *Cblb* knockout mice demonstrated significantly exacerbated severity of experimental autoimmune encephalomyelitis (EAE), augmented T cell infiltration into the central nervous system (CNS) and strongly increased production of GM-CSF in T cells *in vitro* and *in vivo*.GM-CSF neutralization demonstrated that the increased susceptibility of *Cblb*^−/−^ mice to EAE was dependent on GM-CSF. Mechanistically, p50 binding to the GM-CSF promoter and the IL-3/GM-CSF enhancer element “CNSa” was strongly increased in nuclear extracts from Cbl-b-deficient T cells. This study suggests that Cbl-b limits autoimmunity by preventing the pathogenic effects of GM-CSF overproduction in T cells.

## Introduction

The E3 ubiquitin ligase Cbl-b regulates T cell activation thresholds by mediating the requirement for CD28 costimulation (1, 2), thus playing an essential role in immunotolerance and limiting autoimmunity via CD28 (1, 3). Mechanistically, Cbl-b restricts activation of the T cell antigen receptor (TCR) by controlling key molecules involved in T cell stimulation. It has been proposed that Cbl-b inhibits activation of the p85 subunit of phosphoinositide 3-kinase (PI3K) (4, 5), protein kinase C theta (PKCθ), and phospholipase C-γ1 (PLC-γ1) (3, 6) and acts in concert with c-Cbl to promote antigen-induced downregulation of the TCR (7). In addition, Cbl-b represses transactivation of the transcription factor nuclear factor-κB (NF-κB) (8). Furthermore, Cbl-b has been shown to mediate the suppressive effects of TGF-β, leading to reduced T cell sensitivity toward TGF-β and to inhibition by regulatory T cells (9–15).

Cbl-b-deficient mice are prone to develop autoimmunity (1) and two (2, 16) out of three (13) studies showed such mice to have a high susceptibility to experimental autoimmune encephalomyelitis (EAE), the animal model for multiple sclerosis (MS). Along these lines, various genome-wide association studies showed that variants of the CBLB gene are associated with MS (17–19). Furthermore, Cbl-b in T cells was reported to be decreased in patients with MS, and low expression of mRNA was associated with increased risk of relapse (20). The underlying molecular functions of Cbl-b, however, have remained elusive.

In MS and EAE, the central nervous system (CNS) becomes inflamed, and the insulating myelin sheath of axons gets attacked by autoreactive immune cells (21–23). In EAE, the CNS inflammation and subsequent paralysis is induced by active immunization of mice with *self*-antigens such as myelin basic protein (MBP), myelin oligodendrocyte glycoprotein (MOG) or proteolipid protein (PLP) (24). Single epitopes of such proteins, such as MOG35–55 and PLP139–151 peptides, are also sufficient to induce disease (25–27). Onset of MS and EAE is believed to be dependent on autoreactive CD4^+^ T cells which migrate into the CNS to activate and recruit other immune cells (28, 29). Cytokines derived from these CNS antigen-specific CD4^+^ T cells and their corresponding transcription factors are of significant interest in autoimmune research.

Somewhat surprisingly, it has been shown that many proinflammatory cytokines such as IFNγ, IL-12, IL-17A, IL-17F, IL-21, or IL-22 are not required for EAE development (30–38). Instead, it has been shown in several studies that GM-CSF, mainly produced by T cells in an inflammatory setting (39), is an essential cytokine in the pathogenesis of autoimmune neuroinflammation such as EAE (28, 40–42). Its elevated expression in T cells is also implicated in MS (43, 44), and blocking GM-CSF is being tested as a potential treatment option (45).

GM-CSF is a monomeric glycoprotein (46), recognized by a heterodimeric receptor whose β-chain is shared with the IL-3 and IL-5 receptors (47, 48). Signalling at low GM-CSF concentrations occurs via the PI-3 kinase pathway, while higher concentrations further activate JAK2/STAT5 (49) and can lead to proliferation, protection from apoptosis, early commitment to myelopoiesis, differentiation/maturation of committed progenitors and multiple activation and motility functions in mature cells (50). Its ability to promote macrophage polarization and subsequent inflammatory mediator production (51, 52) is of particular interest in the EAE setting.

Along this line of argumentation, IL-3, a cytokine closely related to GM-CSF (47, 48, 53–55), has also been associated with autoimmune neuroinflammation. IL-3 was shown to exacerbate EAE (56) and to be upregulated in MS lesions (57), whereas another study describes it to be a marker of encephalitogenic T helper 1 (T_h_1) and T_h_17 cells but redundant for the severeness of EAE symptoms (58).

Because of the apparent importance of GM-CSF and IL-3 for CNS autoimmunity, their deregulation in MS-patients (44, 57, 59, 60), their functional relationship (47, 48, 55) and the correlation of Cbl-b with autoimmunity as well as the hyper-responsive *Cblb*^−/−^ T cell phenotype (1–3, 17–19), we were interested to find out whether Cbl-b suppresses EAE through the regulation of GM-CSF and IL-3. We show that Cbl-b deficiency leads to massive upregulation of GM-CSF and IL-3 in CD4^+^ T cells, a potentially causative factor in increased neuroinflammation.

## Results

### Cbl-b-deficient T cells produce significantly more GM-CSF and IL-3 *in vitro*

**Figure 1:**
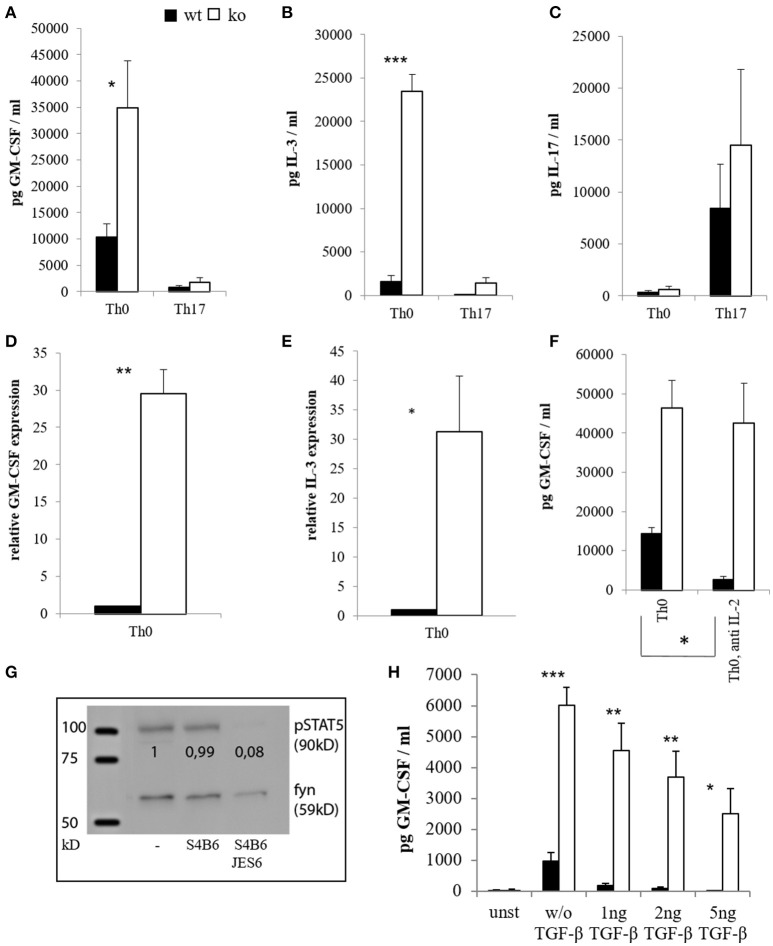
Cytokine expression of CD4^+^ cells *in vitro*. CD4^+^ CD62L^+^ cells of wt and *Cblb*^−/−^ mice were kept in Iscove's Modified Dulbecco's Medium (IMDM) supplemented with 10% FCS, 2 mM l-glutamine and penicillin-streptomycin [50 U/ml] and stimulated with 1 μg/ml anti-CD28 and 3 μg/ml platebound anti-CD3, with or without T_h_17 differentiation conditions (5 ng/ml TGF-β, 20 ng/ml IL-6, 10 ng/ml IL-23, 2 μg/ml antiIFN-γ, 2 μg/ml antiIL-4). GM-CSF [**(A)**, *n* = 4–8; 3–6 independent experiments] and IL-3 levels [**(B)**, *n* = 4–8; 3–6 independent experiments] were measured on day 3 in the cell culture supernatants. To validate T_h_17 differentiation, IL-17 was measured as well [**(C)**, *n* = 4; 4 independent experiments]. RNA was extracted on day 2, and qRT-PCR for GM-CSF [**(D)**, *n* = 6; 5 independent experiments] and IL-3 [**(E)**, *n* = 6; 5 independent experiments] was performed. In some experiments, IL-2 blocking antibodies JES6 (30 μg/ml) and S4B6 (40 μg/ml) were added in combination (= anti IL-2), and GM-CSF levels were measured in the supernatants on day 3 [**(F)**, *n* = 4; 3 independent experiments]. To validate the antibody function, cells were lysed, submitted to western blot and pSTAT5 was detected [**(G)**, 1 out of 2 experiments]. The loading control was considered in the quantification. **(H)** (*n* = 4; 2 independent experiments) shows GM-CSF amounts in supernatants on day 3 of unstimulated (= unst) or stimulated CD4^+^ cells treated with different amounts of TGF-β.

Since T_h_17 cells were implicated in EAE pathogenesis and reported to be an important source of GM-CSF (41, 42, 61, 62), we isolated naïve CD4^+^ T cells from wt and *Cblb*^−/−^ mice, differentiated them into the T_h_17 subset *in vitro* (Figure 1C) and determined their cytokine production. As a result, unskewed T_h_0 cells lacking Cbl-b produced significantly more GM-CSF and IL-3 than their wt counterparts both at protein and mRNA levels (Figures 1A,B,D,E). Interestingly, however, T_h_17 differentiation repressed rather than upregulated GM-CSF and IL-3, and this repression was also pronounced in Cbl-b-deficient T_h_17 cells (Figures 1A,B). Since the downregulation of GM-CSF and IL-3 is dependent on IL-6 [(63) and data not shown], this indicates that the IL-6/STAT3 pathway is not affected by the loss of Cbl-b.

It has been shown that activation of STAT5 induces the expression of GM-CSF and IL-3 (63, 64). It is also known that Cbl-b-deficient T cells produce enhanced amounts of IL-2 (1, 2, 16). To exclude the possibility that increased GM-CSF expression by *Cblb*^−/−^ T cells was just due to auto- and paracrine IL-2/STAT5 signaling, we neutralized IL-2. Blocking IL-2 with the combination of two different antibodies efficiently inhibited STAT5 activation, thus showing this approach to be valid (Figure 1G). As shown in Figure 1F, increased GM-CSF secretion by Cbl-b-deficient T cells was conserved during IL-2 blockade, indicating that this effect is not dependent on IL-2.

The immunosuppressive cytokine TGF-β is implicated in the suppression of EAE (65, 66), and *Cblb*^−/−^ cells have been shown to be relatively resistant against TGF-β-mediated inhibition (9, 10, 15). Therefore, we wanted to find out whether this would also apply to GM-CSF expression. As shown in Figure 1H, TGF-β efficiently suppressed GM-CSF secretion of wt T cells in a dose dependent manner (99% inhibition at 5 ng/ml TGF-β), whereas *Cblb*^−/−^ T cells were partially resistant to TGF-β inhibition (58% inhibition at 5 ng/ml TGF-β). Notably, even with the highest concentration of TGF-β used, Cbl-b-deficient T cells still produced more GM-CSF than wt T cells without addition of TGF-β (Figure 1H).

**Figure 2:**
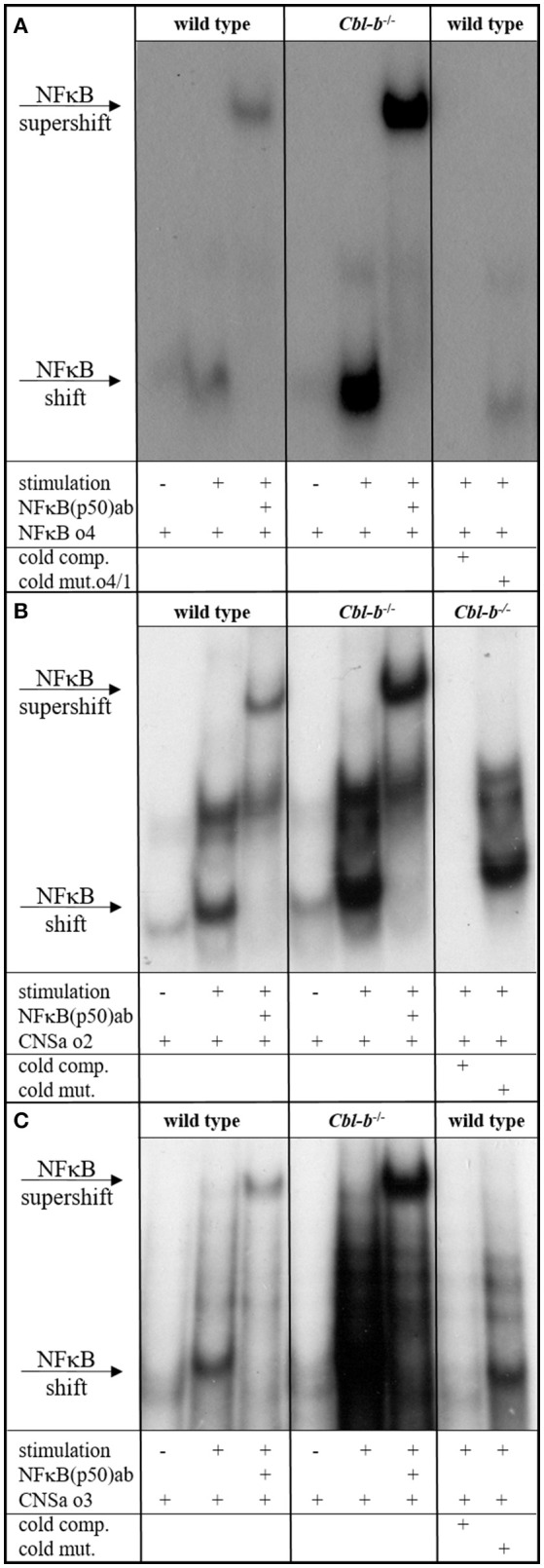
NFκB EMSA. EMSA of nuclear extracts from wt and *Cblb*^−/−^ CD4^+^ T cells, unstimulated or stimulated overnight with 3 μg/ml platebound anti-CD3 and 1 μg/ml anti-CD28. As *wt* oligo, the NFκB consensus sequence within the minimal GM-CSF promoter **(A)** or predicted NFκB sites in the distal enhancer element of the IL-3/GM-CSF gene cluster “CNSa” **(B,C)** were used. To validate binding specificity, mutated oligos were used instead. Wt and mutated sequences were added in excess as unlabeled competition oligos (cold comp., cold mut.). Where indicated, an anti-p50 antibody was added. One representative experiment out of three **(A)** or two **(B,C)** is shown.

The proximal promoter of the GM-CSF gene, encompassing about 120 bp upstream of the transcription start site, contains a CD28 response region that consists of one SP1 and two NF-κB binding sites. The proximal NF-κB site is responsive to TCR signals and binds RelA/p50 heterodimers. Mutation of this site has been shown to reduce the activation of a GM-CSF promoter reporter by at least 50% (67, 68). A distal enhancer element “CNSa” in the IL-3/GM-CSF gene cluster (69) also shows potential NF-κB binding sites. Since our results showed deregulated GM-CSF and IL-3 expression in Cbl-b-deficient T cells, we were interested to find out whether this was reflected by changes in NF-κB binding to the GM-CSF promoter and CNSa. Therefore, we prepared nuclear extracts from anti-CD3/anti-CD28-stimulated wt and *Cblb*^−/−^ CD4^+^ T cells and performed electrophoretic mobility shift assays (EMSA). As DNA oligonucleotides, we used the wt sequence of the proximal NF-κB binding site of the GM-CSF promoter (−110 to −125; Figure 2A) and the sequences of two predicted NF-κB binding sites within CNSa (Figures 2B,C) as well as mutated forms of the respective oligos to confirm the specificity of the interaction. As shown in Figure 2, binding of NF-κB p50 was well inducible by T cell stimulation (validated by a p50 super-shift) at all investigated sites. Notably, p50 binding to the NF-κB binding site was strongly enhanced in nuclear extracts from Cbl-b-deficient T cells at the GM-CSF promoter as well as the CNSa enhancer. Using the mutant oligonucleotide sequences, either labeled (not shown) or as cold competitors, validated the specificity of the NF-κB p50 binding site.

### Cbl-b-deficient mice are hyper-susceptible to EAE which is correlated with dysregulated GM-CSF expression

**Figure 3:**
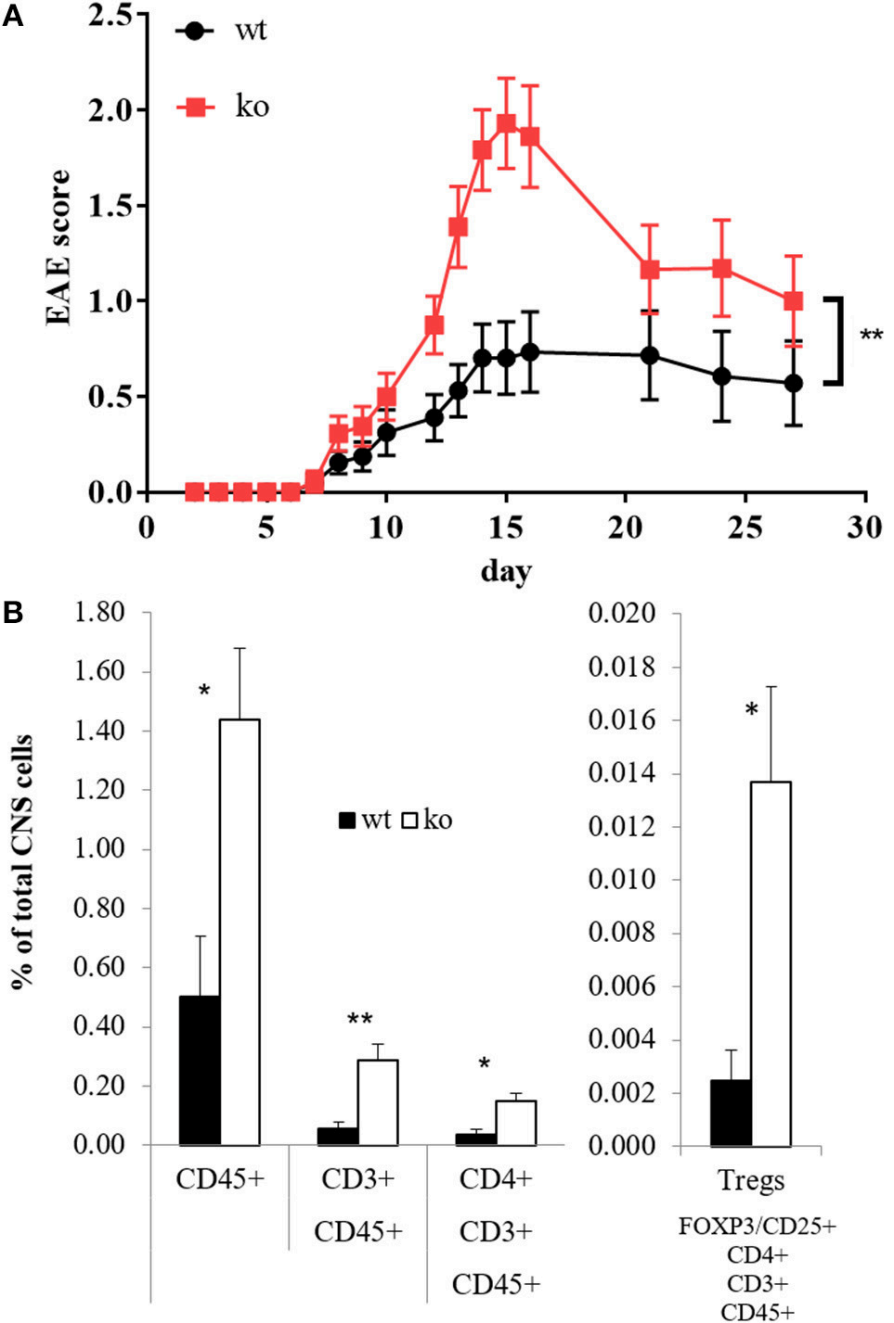
EAE score and CNS-infiltrating T cells. EAE was induced in wt and *Cblb*^−/−^ mice and disease progression was monitored [**(A)**, *n* = 16–18; 5 independent experiments]. At the peak of disease (day 14), FACS analysis of brain and spinal cord was performed [**(B)**, *n* = 6; 2 independent experiments].

In several models, Cbl-b has been shown to be crucial for tolerance induction and prevention of autoimmunity (1, 3, 70, 71). However, the studies on Cbl-b in EAE so far did not yield consistent results (2, 13, 16). Given the fact that Cbl-b is a threshold regulator in T cells, these divergent results could be due to different EAE protocols (1, 2, 72). To address this issue, we used an EAE protocol that leads to only mild signs of disease in wt mice. Applying this protocol, Cbl-b-deficient animals demonstrated significantly enhanced disease severity (Figure 3A), which was accompanied by significantly increased T cell infiltration into the CNS (Figure 3B). The frequency of regulatory T cells (Tregs) was increased as well but was not sufficient to impair EAE progression in *Cblb*^−/−^ animals (Figure 3B, right) likely due to the increased resistance of *Cblb*^−/−^ effector T cells to Treg suppression *in vivo* (9, 13).

**Figure 4:**
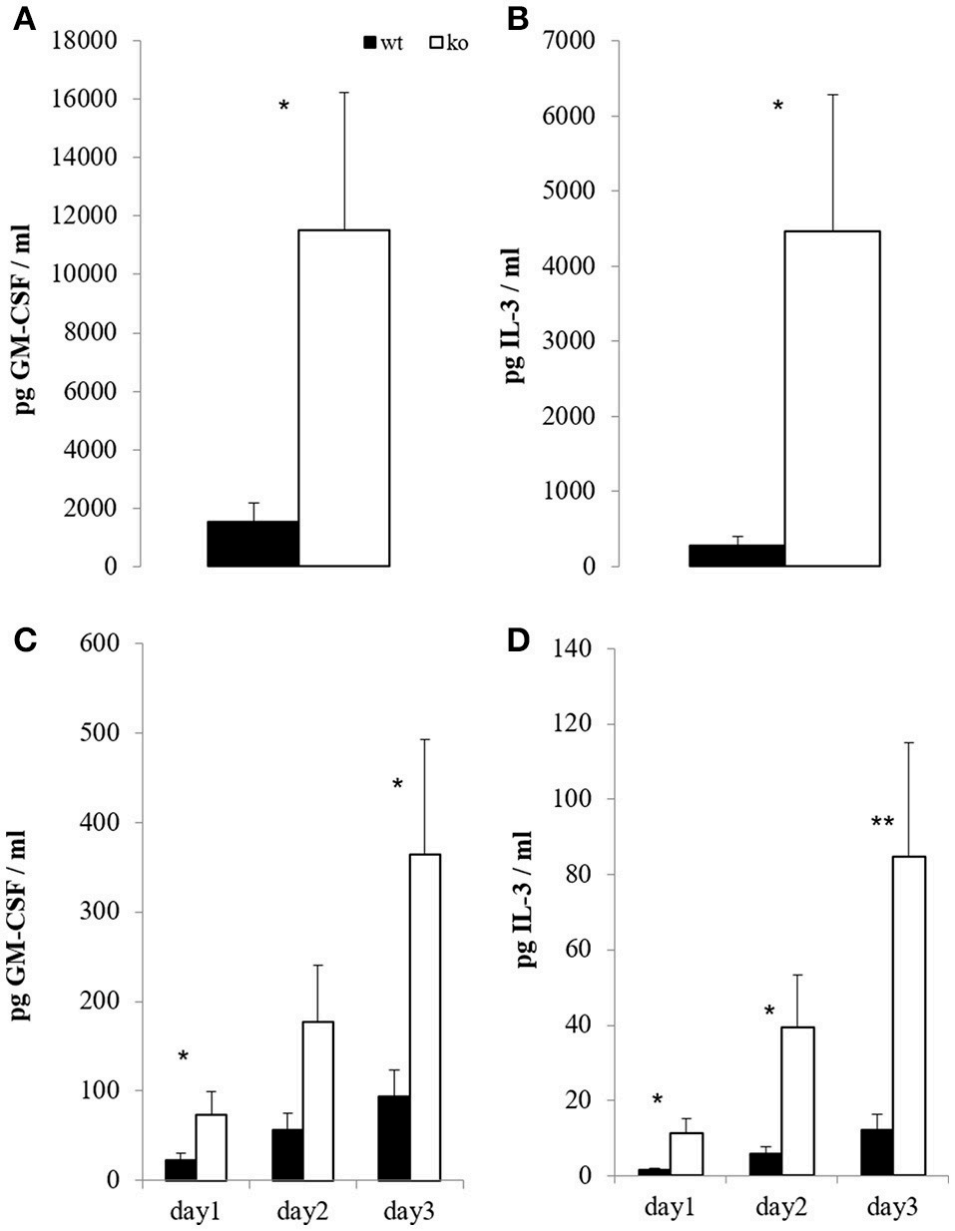
*In vitro* T cell recall. At the peak of EAE (day 14), mononuclear cells from brain and spinal cord were restimulated overnight with 5 μg/ml platebound anti-CD3; GM-CSF [**(A)**, *n* = 6; 2 independent experiments] and IL-3 [**(B)**, *n* = 6; 2 independent experiments] were measured in the supernatants. In parallel, draining inguinal lymph node cells were restimulated with 100 μg/ml MOG for the indicated time periods, and GM-CSF [**(C)**, *n* = 8–10; 3 independent experiments] and IL-3 [**(D)**, *n* = 8–10; 3 independent experiments] were measured in the supernatants.

On the peak of disease, restimulation of mononuclear CNS cells with CD3 crosslinking led to strongly enhanced GM-CSF and IL-3 secretion in the absence of Cbl-b (Figures 4A,B). Consistent with this observation, restimulation of draining lymph node cells with the MOG peptide showed the same effect (Figures 4C,D). Importantly, cells isolated from non-MOG-challenged control mice did not express any of these cytokines upon stimulation (not shown).

To test the relevance of GM-CSF for the increased EAE disease severity of *Cblb*^−/−^ mice we therapeutically administered GM-CSF blocking antibodies to *Cblb*^−/−^ and wt mice during the course of an EAE experiment.

**Figure 5:**
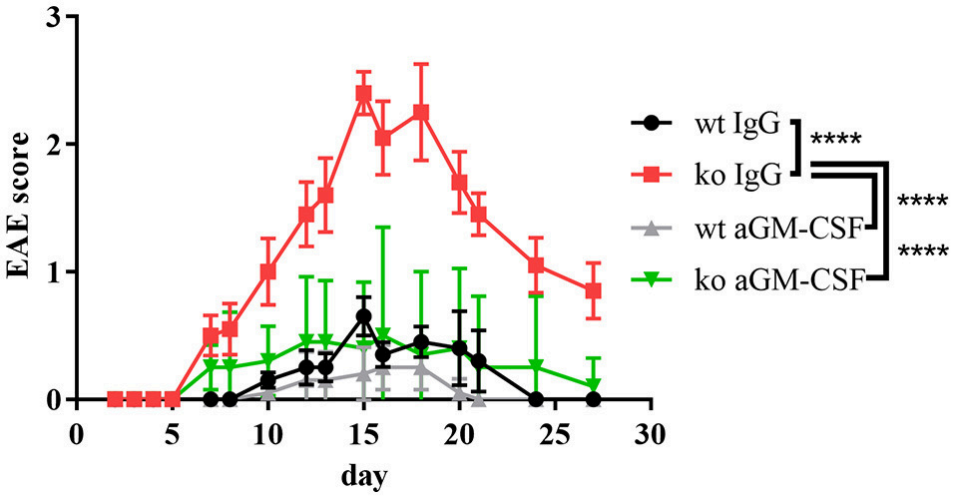
aGM-CSF therapy in EAE. EAE was induced in wt and *Cblb*^−/−^ mice (*n* = 5 per group) and 400 μg aGM-CSF or IgG control antibody were injected i.p. on day 6, 9, 12, 15, 17, and 20. Disease progression was monitored.

Strikingly, neutralization of GM-CSF led to an exorbitant amelioration of EAE symptoms in *Cblb*^−/−^ mice down to wt levels (Figure 5).

Overall, our findings demonstrate that the increased susceptibility of *Cblb*^−/−^ mice to a MOG-induced neuroinflammatory autoimmune reaction *in vivo* is mediated via GM-CSF and suggests that Cbl-b limits autoimmunity by preventing the pathogenic effects of GM-CSF overproduction in T cells.

## Discussion

Initially, GM-CSF was not considered as a prominent cytokine in neuroinflammatory diseases such as EAE or MS. Instead, CNS autoimmunity was long thought to be mediated by T_h_1 and T_h_17 cells through the production of IFNγ and IL-17 until the non-redundant function of T cell-derived GM-CSF in EAE was demonstrated in a number of publications (28, 41, 42); this led to the further discovery that T cells in MS show elevated GM-CSF production (43, 44).

While there is agreement on T cells being the main source for GM-CSF, there is no consensus on the T cell subset that is primarily responsible for the increased production of this cytokine. There are many reports describing T_h_1 or T_h_17 cells as main GM-CSF producers (28, 42), some claim that there is a GM-CSF-producing T cell subset (63) while others do not link GM-CSF production to T cell polarization (41). In this study, stimulation of naïve CD4^+^ T cells with CD3 and CD28 crosslinking without any skewing conditions (T_h_0) yielded the highest levels of GM-CSF, while T_h_17 differentiation clearly repressed GM-CSF production (Figure 1A). This was mainly due to the action of IL-6 (not shown), which is in agreement with previous reports (42, 63). Thus, we show that GM-CSF is not a T_h_17-specific cytokine under *in vitro* differentiation conditions.

The E3 ubiquitin ligase Cbl-b is an established negative regulator of T cell activation, providing a “safety net” against autoimmunity. Here we provide strong experimental evidence that Cbl-b regulates lymphatic GM-CSF expression *in vitro* and *in vivo* during EAE pathogenesis (Figures 1, 4).

We and others have shown that Cbl-b indirectly controls TGF-β-mediated inhibition in T cells (9, 10, 15). The reduced effectiveness of TGF-β in *Cblb*^−/−^ mice and the fact that TGF-β ameliorates EAE (65, 66, 73, 74) potentially explain why *Cblb*^−/−^ mice develop such severe EAE (Figure 3A), probably secondary to increased GM-CSF expression (Figures 1, 4).

Interestingly and along this line of argumentation, GM-CSF expression itself is inhibited by TGF-β and this effect is considerably dampened in *Cblb*^−/−^ CD4^+^ T cells (Figure 1H).

Additionally, as described previously, GM-CSF production is induced by NF-κB binding to the GM-CSF promoter (67, 68, 75). Furthermore, it has been shown that Cbl-b negatively regulates NF-κB (76) and that NF-κB binding to the IL-2 promoter is enhanced in Cbl-b-deficient mice (16). In keeping with these reports, we show that NF-κB binding to the GM-CFS promoter as well as to a distal enhancer element in the IL-3/GM-CSF gene cluster is enhanced in Cbl-b-deficient mice (Figure 2), providing further mechanistic insight into the observed robust upregulation of GM-CSF and IL-3 in Cbl-b-deficient T cells.

The discovery of IL-3 involvement in the development of EAE (56) is even more recent but not unexpected due to its close relationship to GM-CSF (54, 55). Similar to the effect on GM-CSF, the dysregulation of IL-3 in Cbl-b-deficient T cells is also drastic, an observation of potential interest, especially once more research on IL-3 in CNS autoimmunity has been conducted.

Since MS has a family recurrence rate of 20% (77), genetic factors are evolving to be of increasing interest in MS research. Cbl-b seems to be one such genetic factor, as it has emerged to be a potential MS risk gene (17–19). This fits well to our results, which show that Cbl-b-deficient mice develop drastically increased EAE symptoms and CNS infiltration by T cells (Figure 3). As the frequency of Tregs is also increased (Figure 3B) we hereby prove that the higher EAE susceptibility of *Cblb*^−/−^ mice is not due to diminished Treg numbers. We showed not only that more T cells infiltrate the CNS, but also that they produce more GM-CSF and IL-3 upon restimulation with CD3 crosslinking (Figures 4A,B). Furthermore, restimulation of draining lymph node cells with MOG peptide resulted in significantly increased GM-CSF and IL-3 levels in supernatants from *Cblb*^−/−^ cells (Figures 4C,D). As treatment with MOG would only trigger MOG-experienced cells, and CD3 crosslinking alone would only stimulate cytokine production in effector T cells, this shows that in the *Cblb*^−/−^ mice more effector cells produce more GM-CSF and IL-3. This finding may prove to be a useful gauge: low lymphatic Cbl-b expression in MS patients could be an indication for higher levels of GM-CSF in MS lesions and better responses could be expected from antibody therapies. This speculation is strongly supported by our blocking experiments, which showed that neutralization of GM-CSF was particularly effective in Cbl-b-deficient mice (Figure 5). Research on such therapeutic antibodies has already yielded promising results: Blockade of GM-CSF receptor has been reported to ameliorate chronic as well as relapsing-remitting EAE (78), and a monoclonal antibody against human GM-CSF (MOR103) has already been tested in a phase Ib trial for MS (45).

Taken together, we identified the Cbl-b/GM-CSF signaling axis as a potentially causative actor in neuroinflammatory patho-mechanism.

## Materials and methods

### Mice

Cbl-b knockout mice on a C57Bl/6 background have been described previously (1). Mice were maintained under SPF conditions. All animal experiments were performed in accordance with the Austrian “Tierversuchsgesetz” (BGBI. Nr.501/1989 i.d.g.F. and BMWF-66.011/0061-II/3b/2013) and were approved by the Bundesministerium für Wissenschaft und Forschung (bm:wf).

### EAE induction

Wt and *Cblb*^−/−^ mice received s.c. injection of 300 μl emulsion containing 200 μg MOG35-55 (GenScript) in incomplete Freund's adjuvant (ThermoFisherScientific) supplemented with killed Mycobacterium tuberculosis (BD; 2 mg/ml). Pertussis toxin (Sigma; 500 μg in 0.1 ml PBS) was injected intraperitoneally on days 0 and 2. The mice were observed from day 0, and the EAE clinical scores were evaluated every day as follows: 0.5, partial tail limpness; 1, tail limpness; 1.5, reversible impaired righting reflex; 2, impaired righting reflex and hindlimb weakness; 2.5, paralysis of one hindlimb; 3, paralysis of both hindlimbs; 3.5, paralysis of both hindlimbs and one forelimb; 4, hind- and forelimb paralysis; 5, death (79).

### Antibody therapy

Four hundred microgram aGM-CSF (BioXcell BE-0259; clone MP1-22E9) or IgG2a (BioXcell BE0089, clone 2A3) in 400 μl PBS were injected intraperitoneally to all mice, as soon as the first mice showed EAE symptoms on day 6. Treatment was administered on day 6, 9, 12, 15, 17, and 20 after EAE induction.

### Brain and spinal cord homogenization

Mice were perfused with PBS 14 days after EAE induction, and brain and spinal cords were taken and cut into small pieces in 5 ml PBS supplemented with 2.5 mg/ml collagenase (Sigma), 1.8 mg/ml DNAse (Sigma), 2.5 mM MgCl and 2% fetal calf serum (FCS, S1810). After 45 min in a humid, 37° incubator, EDTA was added (final concentration 1 mM) and incubated for another 5 min. The pieces were transferred onto a 70 μm cell strainer and pushed through with the back of a 10 ml syringe. The suspension was further passed through a 40 μm cell strainer and then kept on ice for further analysis.

### Isolation of CNS leukocytes

Mononuclear cells were isolated by Percoll (Sigma) gradient centrifugation from homogenized combined brain and spinal cords as described previously (80).

### Restimulation of draining lymph node cells

The draining inguinal lymph nodes were prepared 14 days after EAE induction, pressed through a metal sieve and restimulated in RPMI+++ (Roswell Park Memorial Institute 1640 medium [PAA; E15-039] supplemented with 10% FCS, 2 mM l-glutamine and penicillin-streptomycin [50 U/ml]) with 100 μg/ml MOG.

### Isolation of T cells

Spleen and lymph nodes (axillary, brachial, inguinal) were mashed through a metal sieve, depleted of erythrocytes using the mouse erythrocyte lysing kit (R&D, WL2000) and CD4^+^ T cells were isolated using the “CD4^+^ T Cell Isolation Kit, mouse” (Miltenyi Biotech 130-104-454) according to the manufacturer's protocol. For T_h_0 and T_h_17, cells were further subjected to positive CD62L selection (Miltenyi, Cat.no.130-093-227).

### Flow cytometry

Homogenized brain and spinal cord cells were incubated with FC-block (BD 553141) for 15 min at 4°, then stained for 20 min at 4°C with specific antibodies (CD8 PerCPCy5.5 [eBiosciences 45-0081-80], CD45 APC [eBiosciences 17-0451-81], CD4 V500 [BD 560782], CD3 PB [Biolegend 100214]) diluted 1:200 in PBS/2% FCS. Data acquisition was performed on a FACSVerse and data analysis was conducted using the FlowJo 10.0.8r1 software.

### Western blot

A 48-well plate was coated with PBS containing 3 μg/ml anti-CD3 (2C11, in-house made) and incubated for 3 h at 37°C. After washing with PBS, 1.5 × 10^6^ CD4^+^ cells per well were cultured overnight in 0.5 ml RPMI+++ and costimulated with 1 μg/ml anti-CD28 (BD 553294) and antibodies against IL-2 (JES-6, 30 μg/ml; S4B6, 40 μg/ml).

Cells were collected, washed once in ice-cold PBS and lysed in 30 μl lysis buffer (5 mM NaP_2_P, 5 mM NaF, 1 mM Na_3_VO_4_, 5 mM EDTA, 150 mM NaCl, 50 mM Tris [pH 7.3], 1% NP-40, aprotinin and leupeptin [50 μg/ml each]) for 30 min on ice. After centrifugation (15,000 g, 4°C), protein lysates were subjected to Western blotting analysis with antibodies against pSTAT5 (cell signaling 9351; 1:1000) and fyn (Santa Cruz-16; 1:1,000). Densitometric analysis was performed using ImageJ.

### Nuclear extracts

5 × 10^6^ CD4^+^ cells were harvested, washed once with cold PBS, (1 min, 9,300 rcf, 4°C) resuspended in 300 μl BufferA (10 mM Hepes pH 7.9; 10 mM KCl; 0.1 mM EDTA; 0.1 mM EGTA; 1 mM DTT; 0.5 mM PMSF) and kept on ice for 15 min. 20 μl 10%NP-40 (final concentration 0.63%) were added, the pellet vortexed (10 s) and washed two times with 300 μl BufferA (5 min, 2300 rcf, 4°C). The supernatant was discarded, 30 μl BufferC (20 mM Hepes pH 7.9; 0.4 M NaCl; 1 mM EDTA; 1 mM EGTA; 1 mM DTT; 1 mM PMSF) added and put on a shaker for 30 min at 4°C. After centrifugation (10 min, 13,000 rcf, 4°C) supernatants were taken and stored at −80°C. Protein concentrations were determined by Bradford assay.

### Gel mobility shift assay

Single-stranded oligonucleotides were synthesized by Eurofins MWG Operon and annealed.

Overnight resting and anti-CD3/anti-CD28 stimulated CD4^+^ T cells from wt or Cbl-b*-* deficient mice were lysed and nuclear extracts were prepared. The following oligonucleotides were used, and the core binding motifs are underlined:

NFκB o4:5′ - TCCACAACTCAGGTAGTTCCCCCGC-3′ (spanning −125 to −110 relative to the transcription start of the mouse GM-CSF gene)
mutated o4/1:5′ -TCCACAACTCTCTTAGTTCCCCCGC-3′
CNSa o2:5′ -GAGAAATACAGGGAATTCCCTACTCTGAG*GATAA*TGG-3′ (predicted by TRANSFAC analysis of CNSa)
CNSa mut o2:5′ - GAGAAATACAG*TT*AATTCC*A*TACTCTGAGGATGGTGG-3′
CNSa o3:5′ -TGGATCTTGATGGGAAATTAAGTGAAGT-3′ (predicted, published NFκB binding site within CNSa) (69)
CNSa mut o3:5′ - TGGATCTTGATGGGCGCTTAAGTGAAGT-3′.

Binding reactions and supershifts were performed for 30 min at 4–8°C using the Binding Buffer B-1 (Active Motif 37480) together with Stabilizing Solution D (Active Motif 37488) containing 4 μg mouse T cell nuclear extracts (Active Motif 36042) and 2 μg of anti NF-κB (p50) (Santa Cruz-1190) antibody.

Binding reactions with the 3 × 10^5^ cpm labeled probe were performed for 20 min at 4°-8°C using Binding Buffer C-1 (Active Motif 37484) together with Stabilizing Solution D (Active Motif 37488). Samples were run on a 4% native polyacrylamide gel in 0.5 × TBE for 3 h at 250 V. For competition assays, 10-fold unlabeled oligonucleotides identical to the radioactive-labeled probes were added to the binding reaction.

### qRT-PCR

5 × 10^5^ CD4^+^ cells were cultured in a 96-flat bottom well for 2 days. RNA was extracted using the RNeasy Mini Kit (Qiagen, Cat.no.74106) according to the manufacturer's protocol. For cDNA synthesis, Omniscript RT Kit (Qiagen, Cat.no.205111), Oligo-dT 15 primer (Promega, Cat.no.C1101) and RNase inhibitor (Promega, Cat.no.N2111) were used according to the manufacturer's protocol.

Real-time PCR was performed using Bio&SELL 5x QPCR MixII (Rox) (BS76.520.5000) and TaqMan probes for the relevant genes on a 7500 FAST Real-Time PCR system. Results were normalized to GAPDH.

### Cytokine measurements

Cell culture supernatants were analyzed via Bio-Plex multianalyte technology (BioRad).

### Statistics

Results are expressed as mean ± standard error of the mean (SEM). Groups were compared using the paired Student's *t*-test. For EAE, two-way Anova with Sidak's *post hoc* test was performed. Data analysis was performed using GraphPad Prism 7.00. Significant differences are indicated as ^*^p ≤ 0.05, ^**^p ≤ 0.01, and ^***^p ≤ 0.001, ^****^p ≤ 0.0001.

## Author contributions

TG and GB conceptualization and supervision. SP performed most of the experimental work and data analysis along with TG, GC, NH-K, KA-S, and RH. SP, TG, and GB writing. All coauthors have read and revised the manuscript.

### Conflict of interest statement

The authors declare that the research was conducted in the absence of any commercial or financial relationships that could be construed as a potential conflict of interest.
